# Supplementary material for: A parietal grid-like code rotates with cognitive maps but lags rapid behavioral transfer
Source: bioRxiv. 2025 Oct 7:2025.10.06.680746. Preprint. [Version 1] doi: 10.1101/2025.10.06.680746 (PMC12632635; doi:10.1101/2025.10.06.680746)
Supplement: Supplement 1 [file NIHPP2025.10.06.680746v1-supplement-1.pdf]

bioRxiv preprint doi: https://doi.org/10.1101/2025.10.06.680746; this version posted October 7, 2025. The copyright holder for this preprint (which was not certified by peer review) is the author/funder. This article is a US Government work. It is not subject to copyright under 17 USC 105 and is also made available for use under a CC0 license.

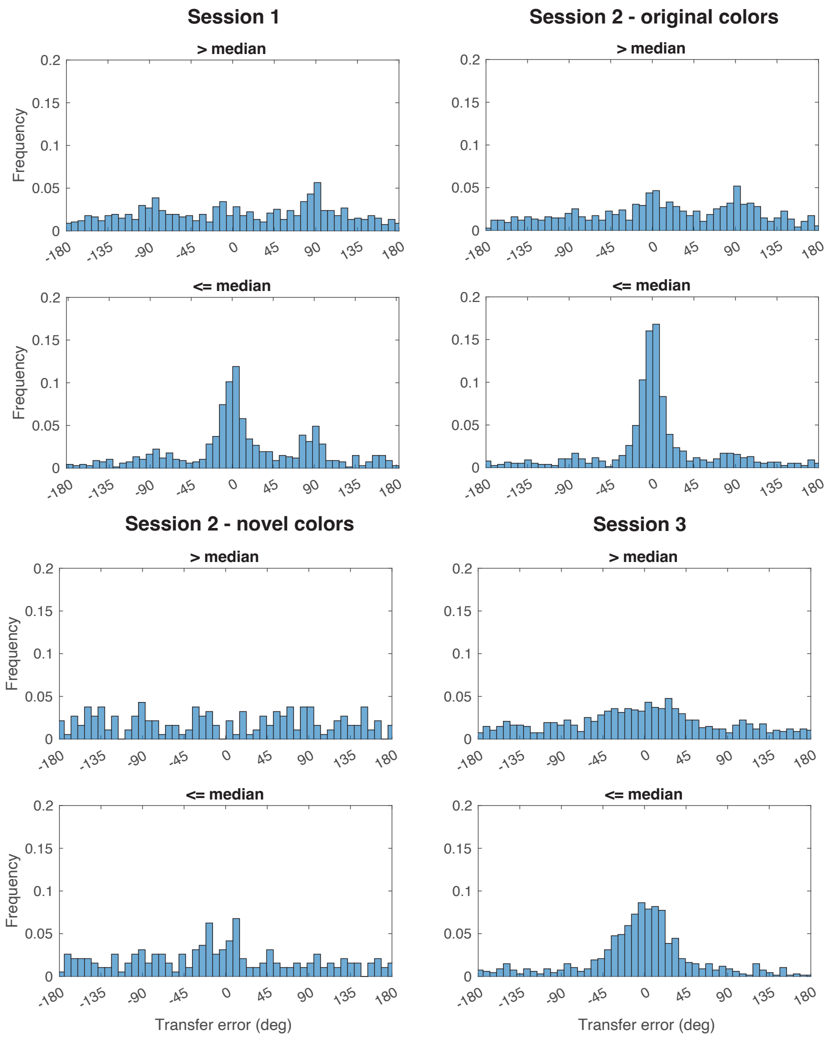

Supplementary Figure 1. Histograms showing angular error on transfer trials measured in degrees across sessions and conditions. Participants have been separated based on a median split of the average transfer error in each condition. Note the peak centered on zero degrees for the top half of participants (i.e. <= median transfer error) in the novel color condition in session 2.

bioRxiv preprint doi: https://doi.org/10.1101/2025.10.06.680746; this version posted October 7, 2025. The copyright holder for this preprint (which was not certified by peer review) is the author/funder. This article is a US Government work. It is not subject to copyright under 17 USC 105 and is also made available for use under a CC0 license.

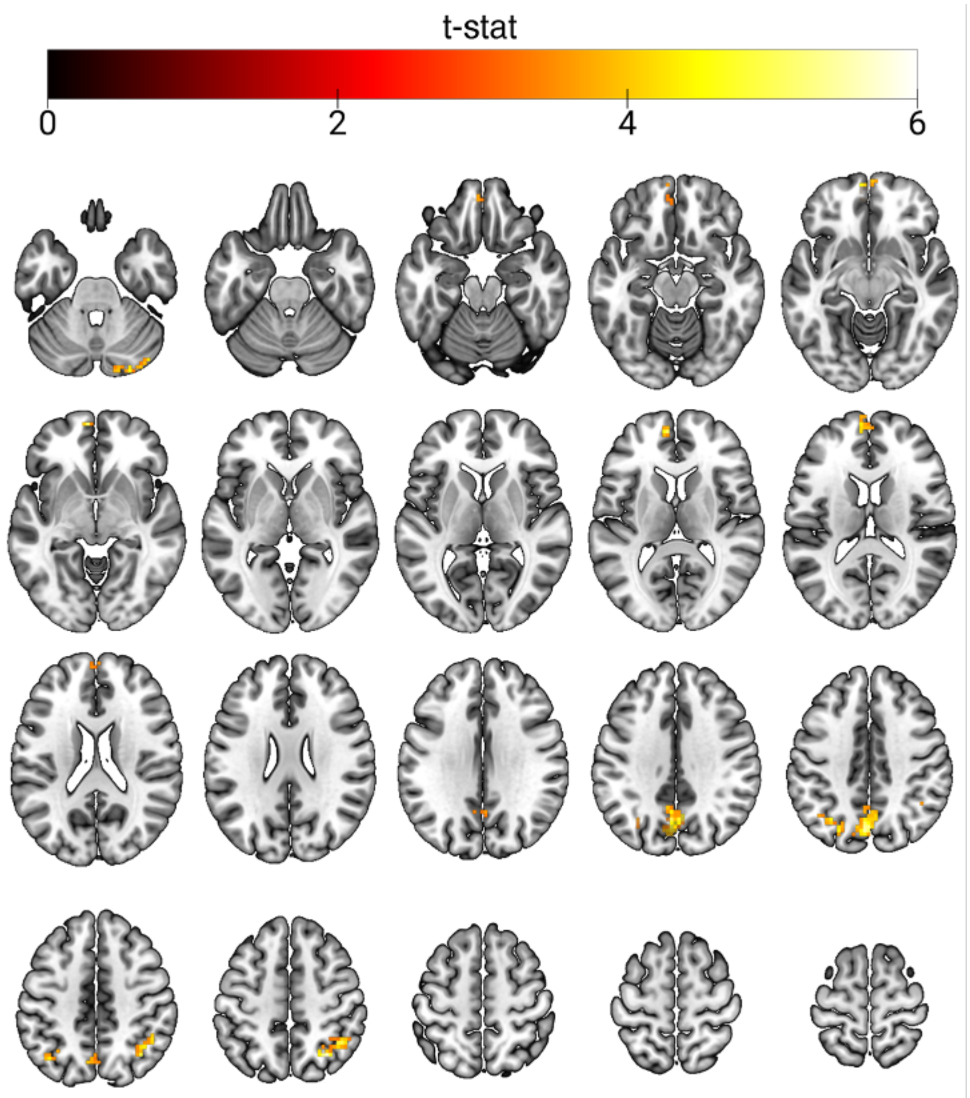

Supplementary Figure 2. Statistical map from whole brain analysis identifying regions responding to a hexagonally symmetrical signal during the response period. Results are displayed at a cluster forming threshold of P<0.001 and corrected for multiple comparisons with permutation tests for defining a cluster extent threshold at P<0.05 (K = 52).

bioRxiv preprint doi: https://doi.org/10.1101/2025.10.06.680746; this version posted October 7, 2025. The copyright holder for this preprint (which was not certified by peer review) is the author/funder. This article is a US Government work. It is not subject to copyright under 17 USC 105 and is also made available for use under a CC0 license.

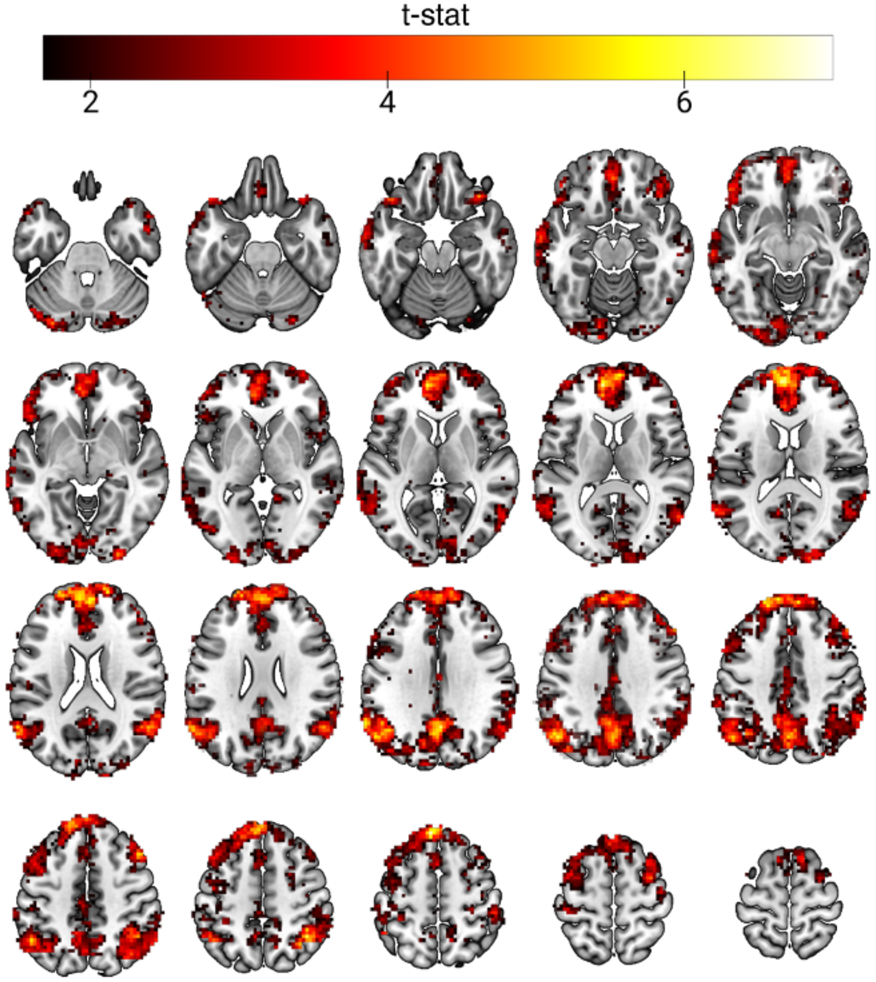

Supplementary Figure 3. Statistical map from whole brain analysis identifying regions responding to a hexagonally symmetrical signal at an uncorrected threshold of $P < 0.05$.

bioRxiv preprint doi: https://doi.org/10.1101/2025.10.06.680746; this version posted October 7, 2025. The copyright holder for this preprint (which was not certified by peer review) is the author/funder. This article is a US Government work. It is not subject to copyright under 17 USC 105 and is also made available for use under a CC0 license.

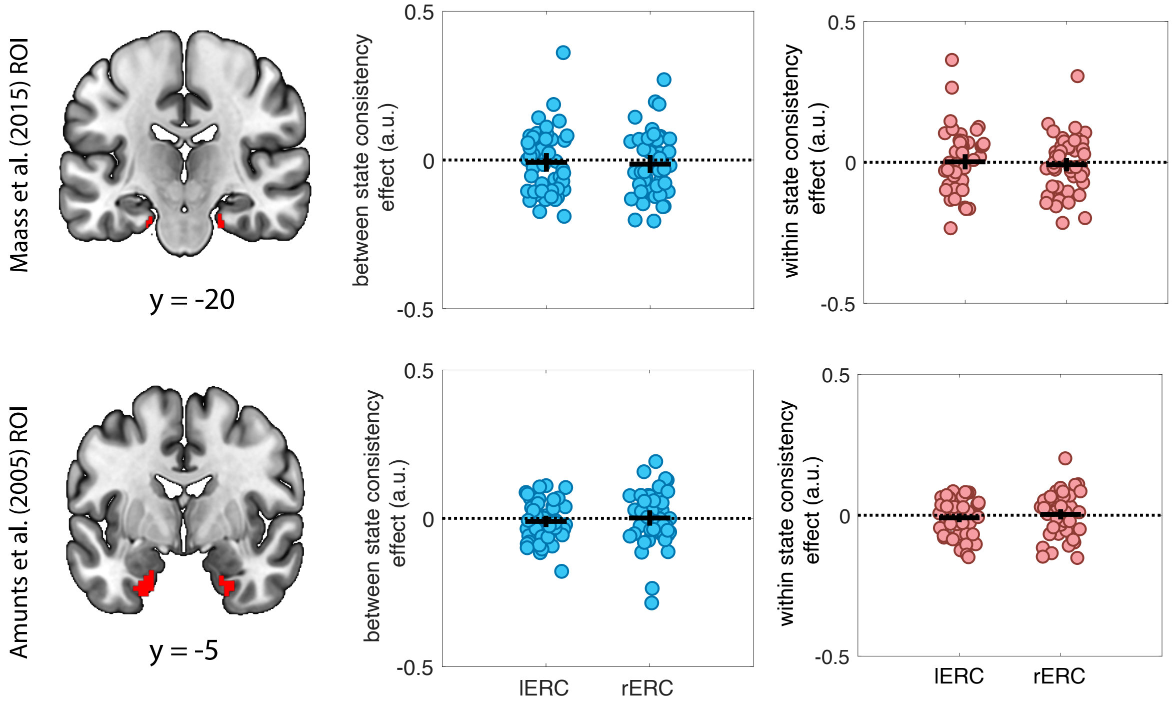

Supplementary Figure 4. Tests for between- and within-state grid angle consistency in two pairs of *a priori* entorhinal cortex ROIs in the left and right hemisphere (lERC, rERC) in which other fMRI studies that have identified grid-like codes (Bao et al., 2019; Park et al., 2021; Raithel et al., 2023). These ROIs are shown on the left-hand side. Each circle represents a participant. Horizontal bars represent the mean, vertical bars represent the 95% CIi. There were no significant results in either set of ROIs.

bioRxiv preprint doi: https://doi.org/10.1101/2025.10.06.680746; this version posted October 7, 2025. The copyright holder for this preprint (which was not certified by peer review) is the author/funder. This article is a US Government work. It is not subject to copyright under 17 USC 105 and is also made available for use under a CC0 license.

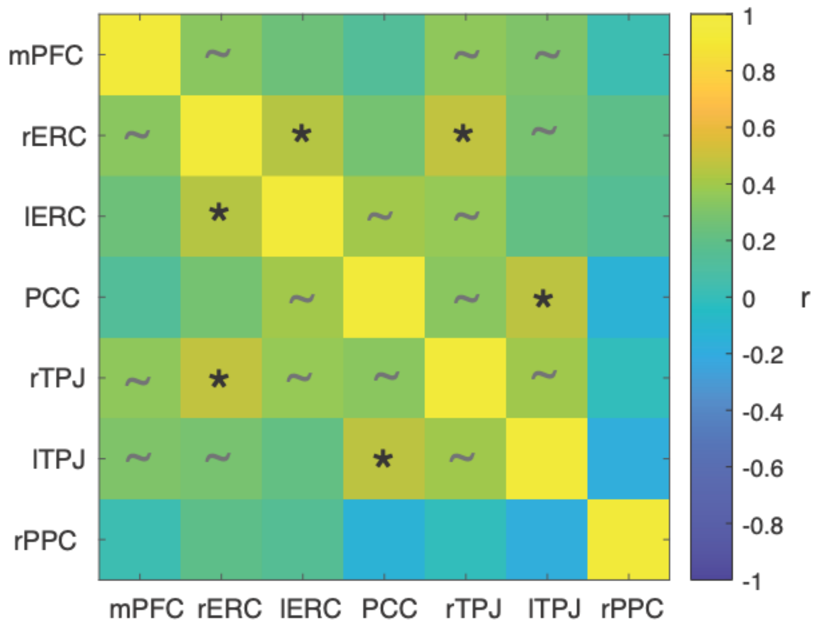

Supplementary Figure 5. Pearson correlation matrix for between-state grid coefficients for different regions of interest. We identified significant positive correlations between these coefficients in the right and left ERC, and between the right temporoparietal junction (TPJ) and right ERC, and between the posterior cingulate cortex and left TPJ (r's(46) ≥ 0.44, P < 0.05, corrected for multiple comparisons). Coefficients in other regions identified in the hexagonal symmetry analysis were also weakly positively correlated, though these correlations did not survive multiple comparisons correction. Notably, these correlations were weakest or trended in a negative direction between these other ROIs and the rPPC, which was the only brain region with grid representations that had a consistent phase relationship across rotation states. Medial prefrontal cortex (mPFC), entorhinal cortex (ERC), posterior cingulate cortex (PCC), tempro-parietal junction (TPJ), posterior parietal cortex (PPC) r = right, l = left. * P < 0.05, corrected for multiple comparisons, ~ P < 0.05 uncorrected.

bioRxiv preprint doi: https://doi.org/10.1101/2025.10.06.680746; this version posted October 7, 2025. The copyright holder for this preprint (which was not certified by peer review) is the author/funder. This article is a US Government work. It is not subject to copyright under 17 USC 105 and is also made available for use under a CC0 license.

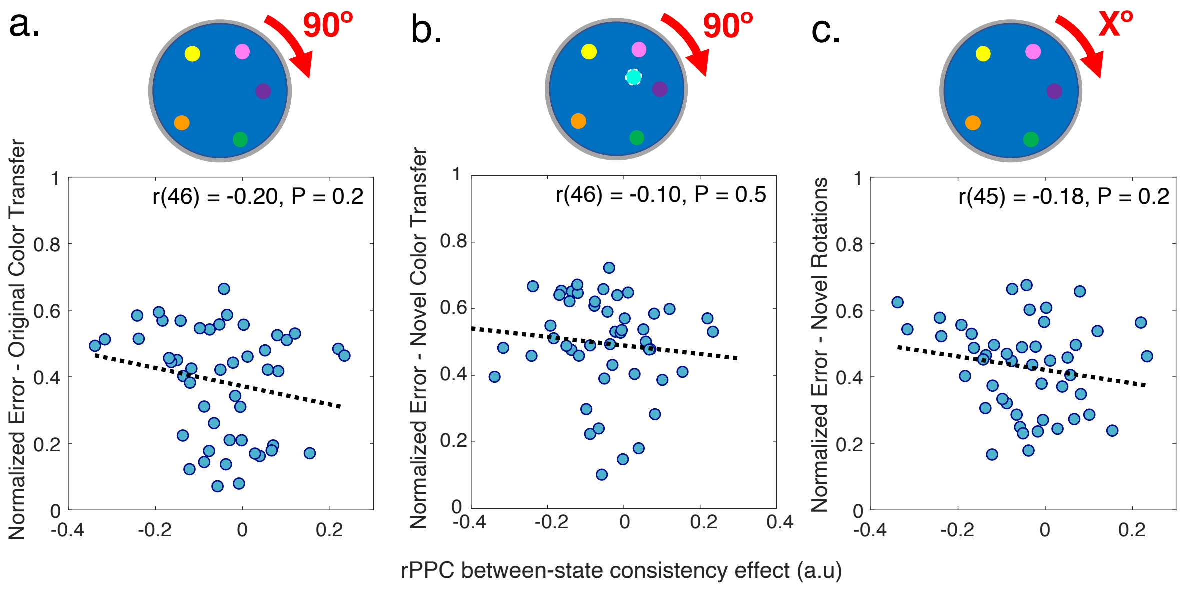

Supplementary Figure 6. Scatterplots between right posterior parietal cortex (rPPC) between-state grid-like code consistency and normalized error on transfer trials for original colors (**a**) and novel colors (**b**) in session 2, and novel rotations in session 3 (**c**).

bioRxiv preprint doi: https://doi.org/10.1101/2025.10.06.680746; this version posted October 7, 2025. The copyright holder for this preprint (which was not certified by peer review) is the author/funder. This article is a US Government work. It is not subject to copyright under 17 USC 105 and is also made available for use under a CC0 license.

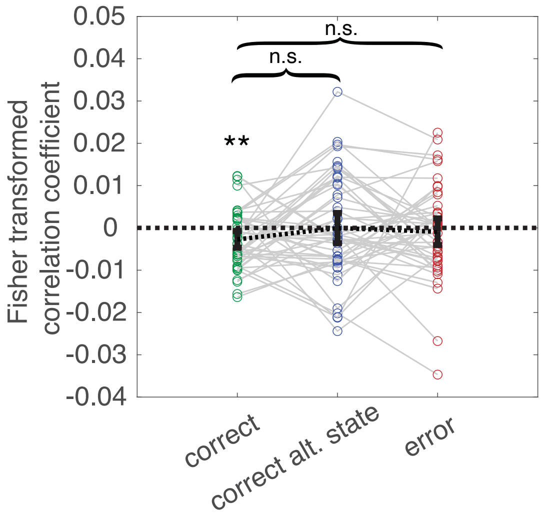

Supplementary Figure 7. Trial-wise coefficients for between state grid-like code phase consistency organized by trial type**.** We expected that if this cognitive grid-like code in rPPC reflects participants belief about the task state, then the grid angles should be anti-phase between states on trials where they respond correctly given the current state (i.e. more negative coefficients), and would be in-phase between states in trials where participants responded as though they were in the alternate state. On correct trials, where participants were within 45 degrees of the target, there was a significant negative tendency for these coefficients, two-tailed one-sample t-test: t(47) = 2.76, P = 0.008, d = 0.40), as expected. However, coefficients on trials that were correct for the alternate state (i.e. within 45 degrees of the target location in the alternate state) were not significantly different from zero (Figure 5a; t(47) = -0.02, P = 0.9, d = 0.003). There were also no significant differences between coefficients on correct trials and trials correct for the alternate state (t(47) = 1.36, P = 0.18, d = 0.20), or between correct trials and other kinds of error trials (t(47) = 0.96, P = 0.34, d = 0.14).

bioRxiv preprint doi: https://doi.org/10.1101/2025.10.06.680746; this version posted October 7, 2025. The copyright holder for this preprint (which was not certified by peer review) is the author/funder. This article is a US Government work. It is not subject to copyright under 17 USC 105 and is also made available for use under a CC0 license.

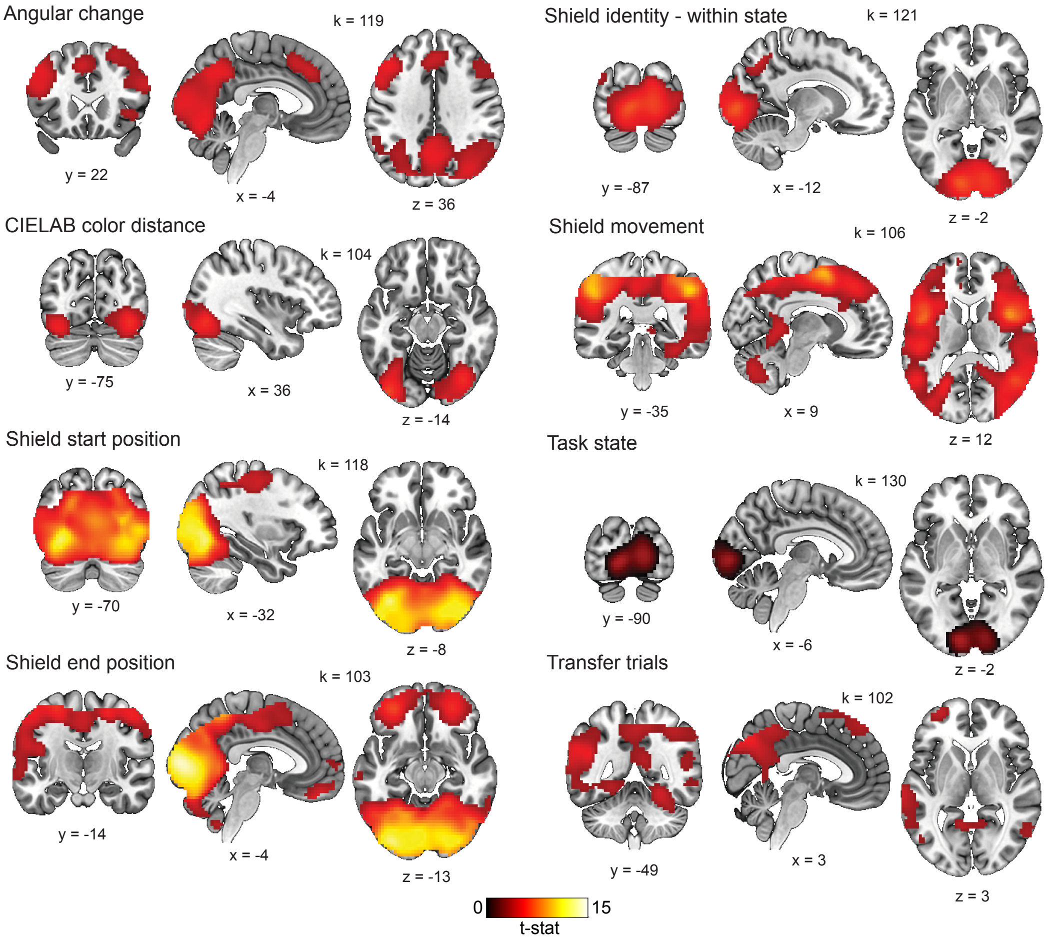

Supplementary Figure 8. Results of whole-brain representational similarity searchlight analysis for all task and behavior related regressors with statistical effects that survived multiple comparisons correction. All statistical maps were defined with a cluster forming threshold of $p<0.001$ and corrected for multiple comparisons with permutation tests for defining a cluster extent threshold at $p<0.05$. Cluster extent threshold for each contrast is given by the value of k. Covariate analyses restricted to voxels that showed a significant effect for the transfer RDM at a whole-brain cluster corrected threshold did not reveal any significant correlations with normalized error on transfer trials for original or novel colors. We also did not find any regions with a significant representation of the invariant positions of each shield color, even at a more liberal threshold of $P < 0.01$.

bioRxiv preprint doi: https://doi.org/10.1101/2025.10.06.680746; this version posted October 7, 2025. The copyright holder for this preprint (which was not certified by peer review) is the author/funder. This article is a US Government work. It is not subject to copyright under 17 USC 105 and is also made available for use under a CC0 license.

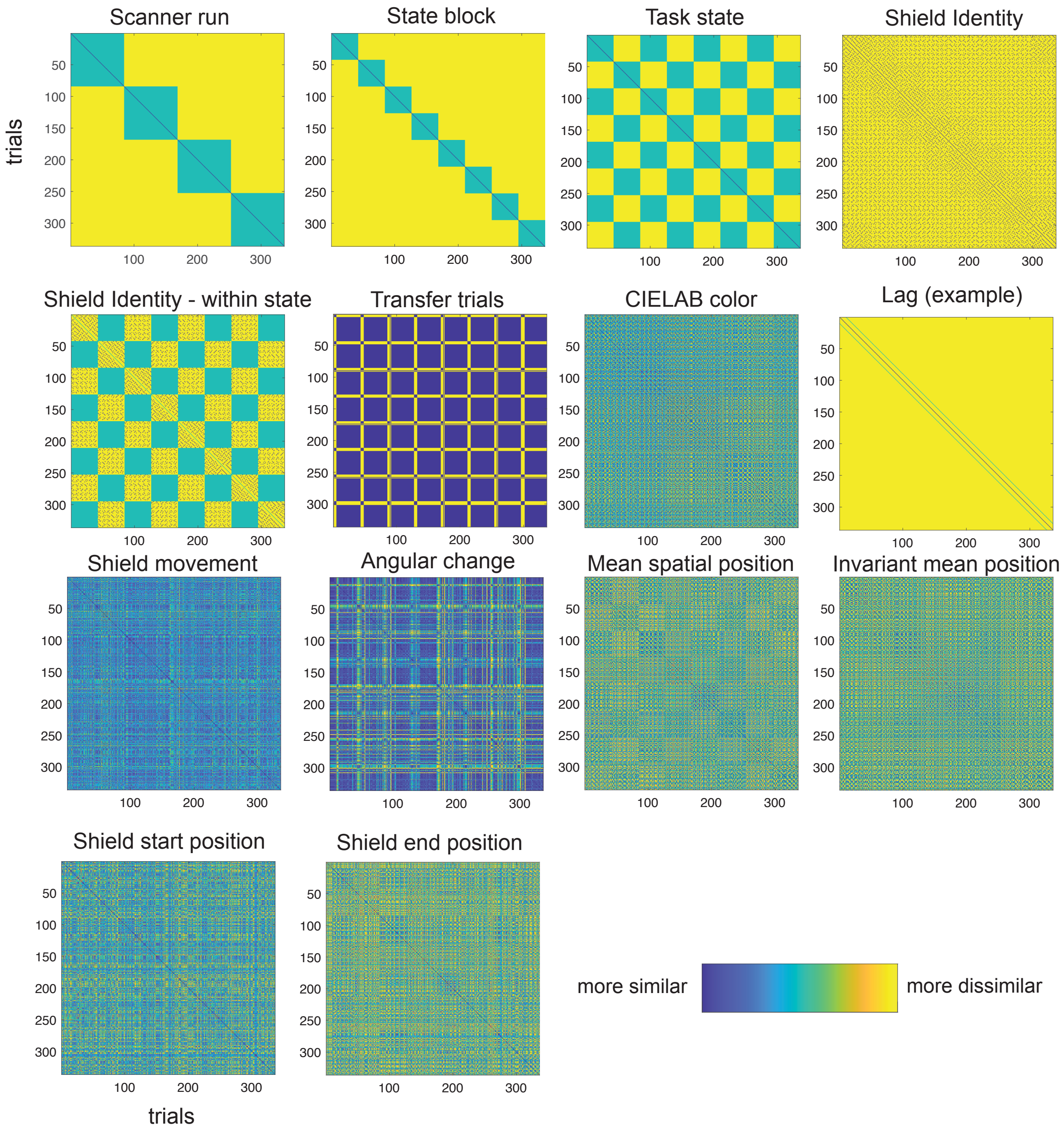

Supplementary Figure 9. Hypothesis trial-wise representational dissimilarity matrices (RDMs) for representational similarity analysis from one randomly chosen example participant.

bioRxiv preprint doi: https://doi.org/10.1101/2025.10.06.680746; this version posted October 7, 2025. The copyright holder for this preprint (which was not certified by peer review) is the author/funder. This article is a US Government work. It is not subject to copyright under 17 USC 105 and is also made available for use under a CC0 license.

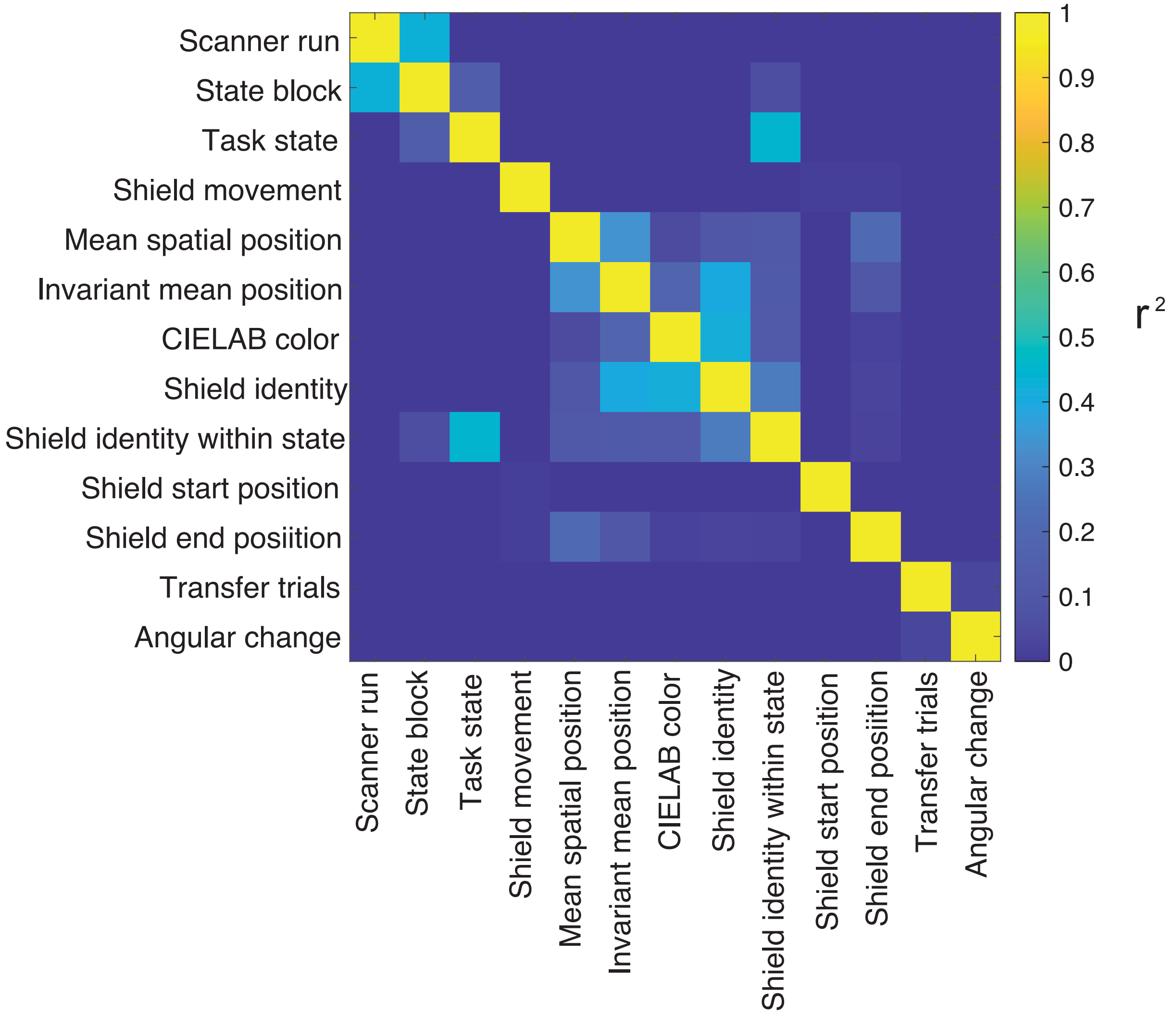

Supplementary Figure 10. Variance shared (Pearson's r^2) between regressors included in representational similarity analysis, excepting lag regressors.

bioRxiv preprint doi: https://doi.org/10.1101/2025.10.06.680746; this version posted October 7, 2025. The copyright holder for this preprint (which was not certified by peer review) is the author/funder. This article is a US Government work. It is not subject to copyright under 17 USC 105 and is also made available for use under a CC0 license.

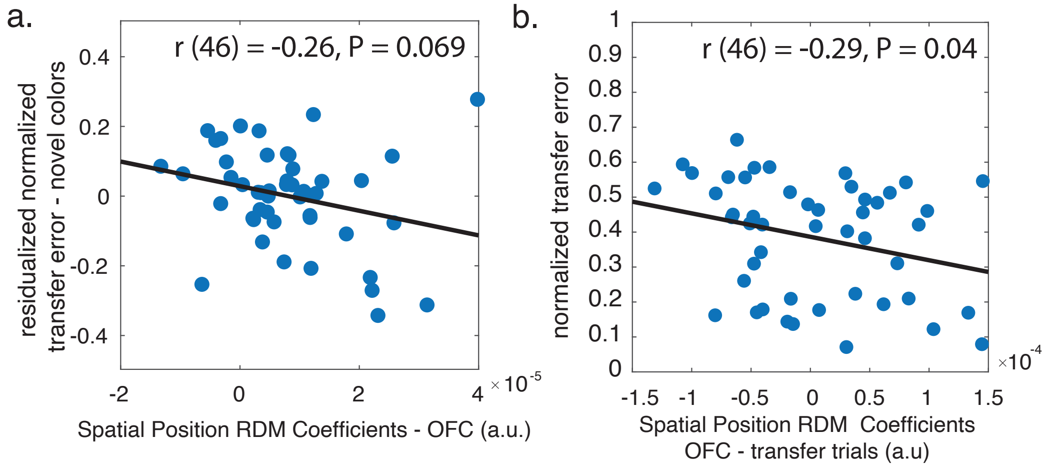

Supplementary Figure 11. Additional tests of relationship between behavior and spatial position representation in bilateral orbitofrontal cortex (OFC). **a.** Scatterplot showing relationship between coefficients for mean spatial position representational dissimilarity matrix (RDM) in a ROI of OFC and normalized error on novel color transfer trials, after statistically removing the linear relationship between normalized error on these trials and normalized error on repeat trials for the original colors. **b.** Relationship of mean spatial position RDM coefficients in OFC for original color transfer trials plotted against normalized transfer error for the same.
